# Personalized Adipofascial Flap: A Game-Changer for Post-Traumatic Ulnar Nerve Neuropathy at the Wrist and Elbow

**DOI:** 10.3390/jpm15110521

**Published:** 2025-11-01

**Authors:** Alessandro Greco, Martina Bizzarri, Lucian Lior Marcovici, Alessia Pagnotta

**Affiliations:** 1 Jewish Hospital of Rome, 00148 Rome, Italy; l.marcovici@ospedaleisraelitico.it (L.L.M.); a.pagnotta@ospedaleisraelitico.it (A.P.); 2 Department of Orthopaedics and Traumatology, Sapienza University of Rome, 00185 Rome, Italy

**Keywords:** ulnar nerve, adipofascial flap, ulnar nerve palsy, post-traumatic neuropathy, peripheral nerve coverage, vascularized adipofascial flap, nerve compression, neurolysis

## Abstract

**Introduction:** Post-traumatic and post-surgical ulnar nerve neuropathies at the elbow and wrist remain challenging conditions often associated with significant sensory and motor impairment. Traditional approaches such as neurolysis alone may be insufficient, especially in complex or recurrent cases. Adipofascial flaps have shown promising outcomes in peripheral nerve surgery. The aim of this study was to evaluate the outcomes of 13 patients with severe ulnar neuropathies who were treated with a size- and shape-personalized adipofascial flap for nerve coverage. **Materials and Methods**: We retrospectively analyzed 13 patients treated between May 2020 and May 2024 for severe post-traumatic or post-surgical ulnar neuropathies. All underwent surgical decompression, external neurolysis, and adipofascial flap coverage. Pre- and postoperative outcomes were assessed with clinical and neurological evaluations and using the QuickDASH and NRS pain scores. **Discussion**: All patients showed improvement in pain and sensory-motor function, including those with complications, supporting the role of flap coverage in neuroprotection. This is the first study to describe the use of adipofascial flaps for pseudo-palsy and painful neuroma-in-continuity of the ulnar nerve at the elbow and wrist level. **Conclusions**: Adipofascial flaps represent a safe, technically feasible, and effective option in complex ulnar nerve injuries, providing both mechanical and biological support. Despite the small cohort, the results suggest strong clinical potential across varied injury patterns.

## 1. Introduction

Post-traumatic ulnar nerve neuropathies may result from a variety of causes, including blunt trauma, penetrating injuries, or as complications following surgical procedures involving the elbow, forearm, or wrist [1,2,3,4,5,6,7]. Ulnar nerve palsy or pseudo-palsy can significantly impair the function of the upper limb, particularly affecting fine motor control of the hand [8,9,10]. Historically, the surgical treatment for severe post-traumatic ulnar neuropathies has consisted of simple nerve decompression at the site of injury [11,12,13,14]. However, outcomes have often been suboptimal in complex, recurrent, or post-traumatic cases [5,15]. More recently, the use of adipofascial flaps for peripheral nerve coverage has demonstrated promising functional results across various pathological conditions [16,17,18,19,20,21,22,23,24,25]. Adipofascial flaps provide a well-vascularized, pliable coverage that closely resembles the native fatty tissue surrounding peripheral nerves. This biological environment may enhance axonal regeneration while reducing the risk of perineural fibrosis, painful neurodesis and finally recurrent neuropathy [17,18,20,26,27,28]. While adipofascial flaps have demonstrated efficacy in the management of recurrent ulnar compression neuropathy at the elbow [17,18,20], the literature remains limited regarding their application in severe post-traumatic or post-surgical cases involving the ulnar nerve at the elbow and wrist [25,29,30]. The aim of this study was to retrospectively evaluate the outcomes of 13 patients with severe sensory-motor ulnar neuropathies at the level of the elbow, forearm, and wrist, treated with adipofascial flap nerve coverage. To our knowledge, this is the first clinical study documenting the utility of this technique in the management of complex post-traumatic ulnar nerve injuries.

## 2. Materials and Methods

**Figure 1:**
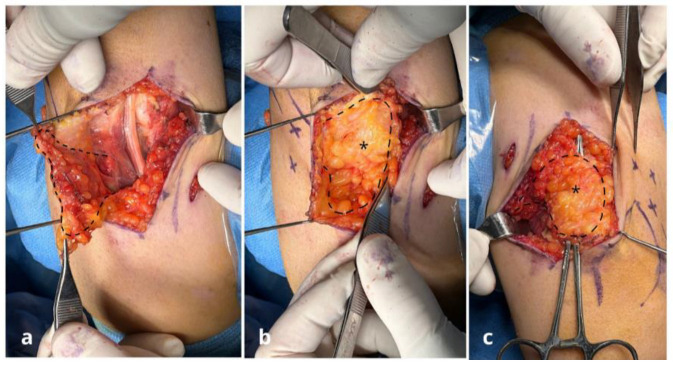
Case n°11. Ulnar nerve neuropathy in a patient with post-traumatic elbow stiffness following radial head fracture-dislocation (patient no. 11). (**a**) After external neurolysis, the ulnar nerve appears swollen and compressed proximal to Osborne’s retinaculum. (**b**) Nerve coverage using a personalized freestyle adipofascial flap (*) based on perforating branches of the posterior ulnar recurrent artery. (**c**) Final flap (*) suturing and intraoperative testing confirm a tension-free nerve, adequately decompressed and free from residual compression.

**Figure 2:**
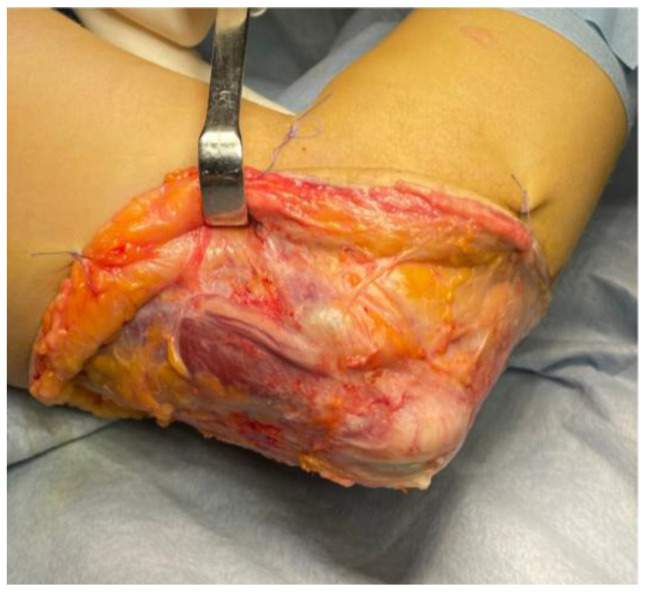
Case n°3. Intraoperative view showing severe stenosis of the ulnar nerve at the epitrochleo-olecranon region. The focal compression led to marked impairment of vasa nervorum circulation, with evident vascular stasis along the nerve.

**Figure 3:**
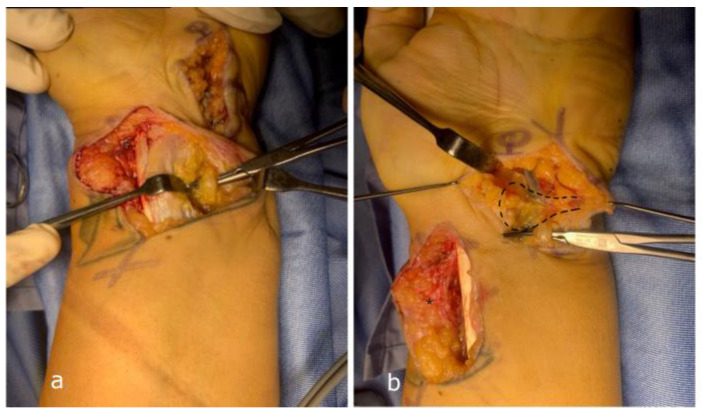
Case n°13. Left wrist with severe ulnar neuropathy and perineural fibrosis secondary to previous temporary percutaneous K-wire fixation for a distal radius fracture (**a**). External neurolysis of the ulnar nerve was performed from proximal to distal with meticulous removal of adhesions, followed by coverage with a local freestyle adipofascial flap (*) (**b**).

Between May 2020 and May 2024, we retrospectively observed a total of 131 patients who underwent surgical treatment around the elbow and wrist in which the ulnar nerve was decompressed. We selected 86 patients in whom, at the end of the surgical procedure, the nerve was covered by a vascularized adipofascial flap. From these patients, we selected only patients who underwent surgical treatment for severe post-traumatic or post-surgical impairment of the ulnar nerve, diagnosed as either neurapraxia or axonotmesis. A total of 13 patients were included in this study. All patients presented with significant sensory deficits (numbness or paresthesia) in the ulnar side of the hand (typically the fourth and fifth fingers), and/or motor symptoms such as intrinsic hand muscle weakness or atrophy. In each case, the ulnar nerve was surgically decompressed, released from surrounding scar tissue, and subsequently covered with a size and shape-personalized adipofascial flap (Table 1). A standard x-ray of the elbow or wrist was performed in all patients before surgery. At least 40 days following nerve injury, all patients underwent electromyographic and nerve conduction studies. These evaluations were performed to confirm the site of nerve compression and to assess the severity of ulnar nerve injury. In all cases, findings were consistent with neurapraxia or axonotmesis of the ulnar nerve. Pre-operative assessments included the Numerical Rating Scale (NRS) for pain and the Quick Disabilities of the Arm, Shoulder, and Hand (Q-DASH) questionnaire, which were administered both before surgery and at a minimum of one-year postoperative follow-up. All patients underwent a thorough clinical and neurological examination. Sensory function was assessed in all patients by testing cutaneous sensitivity in the fourth and fifth digits. Motor function was evaluated in all patients through assessment of intrinsic hand muscle tropism and manual strength testing, including the Froment’s sign, as a clinical indicator of ulnar nerve-related muscle weakness. Tinel’s sign was elicited along the entire course of the ulnar nerve both preoperatively and at each postoperative follow-up. This study was conducted in accordance with the principles of the Declaration of Helsinki. Written informed consent was obtained from all patients involved in the study. In all 13 patients, a freestyle adipofascial flap was used to cover the ulnar nerve (Figure 1). The type and design of the flap were tailored according to the specific local anatomical and clinical conditions of each patient, rather than applying a standardized approach. In 7 cases, the ulnar nerve was damaged at the elbow level. The injury site was located in the posterior region of the elbow, to the cubital tunnel (epitrochleo-olecranon groove). The stenosis and/or neuroma-in-continuity was found slightly proximal to, or underneath, Osborne’s retinaculum (Figure 2). In 6 cases, the nerve injury was located at the wrist level. In these patients, the injury site was in the anterior wrist region, corresponding to or proximal to the Guyon’s canal. In all cases, external neurolysis was initiated proximal to the site of injury, as indicated by clinical and electromyographic evaluation. The ulnar nerve was decompressed from proximal to distal along the course of the lesion (Figure 3), then definitively positioned in its most appropriate anatomical location, and subsequently covered with a personalized freestyle adipofascial flap.

### Surgical Techniques

All patients underwent general anesthesia and an axillary locoregional block. A standard tourniquet or a disposable sterile elastic exsanguination tourniquet (HemaClear, OHK Medical) was applied to exsanguinate the arm. All patients were positioned supine with the upper limb supported on a surgical hand and arm (Table 1).

**Figure 4:**
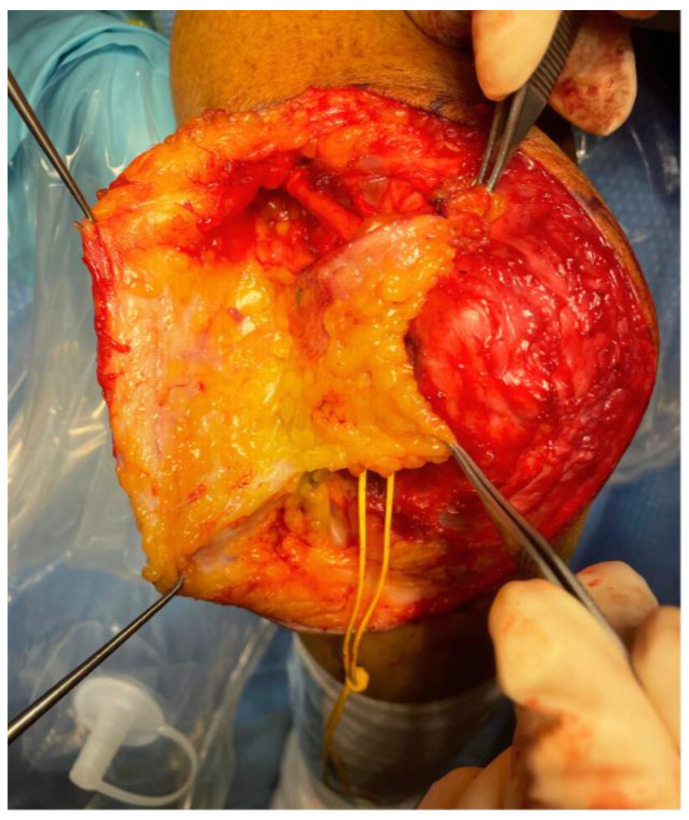
Case n°10. Freestyle anteromedial adipofascial flap used to achieve complete coverage of the ulnar nerve after anterior transposition. The abundant adipose tissue provides effective protection of the nerve, which in thin patients is otherwise highly exposed to trauma, often resulting in a positive Tinel’s sign at the site of transposition.

**Figure 5:**
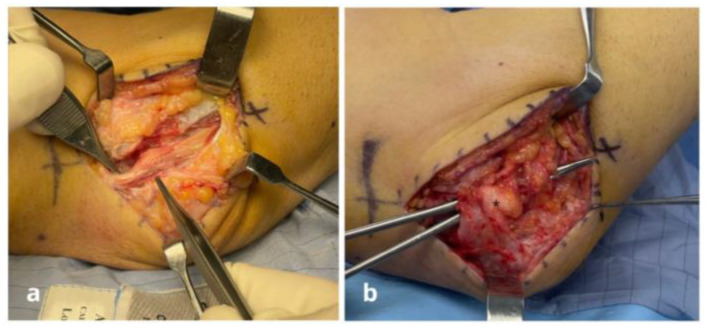
Case n°9. Ulnar nerve entrapment with fascicular exposure at the level of the cubital tunnel (**a**). Nerve coverage following external neurolysis using a freestyle adipofascial flap (*) (**b**).

**Figure 6:**
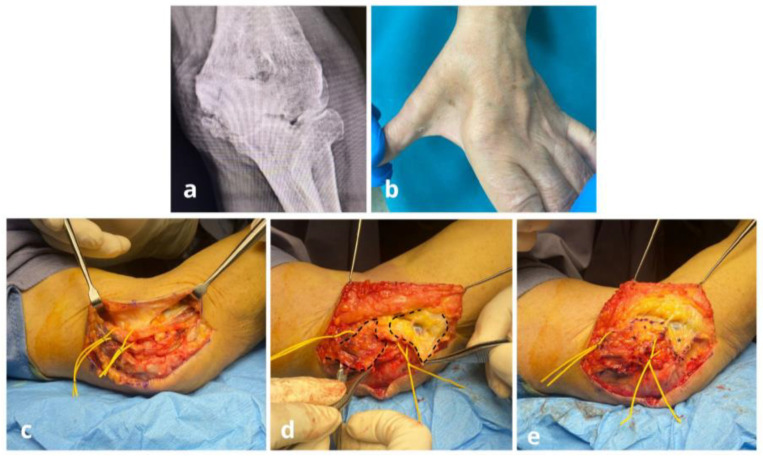
Case n°7. Anteroposterior radiograph of the left elbow in a patient with multiple hereditary exostoses, showing osteophytes in the posteromedial region of the joint (**a**). Clinically, the patient exhibited severe ulnar nerve impairment, with atrophy of the first web space and claw-hand deformity (**b**). The ulnar nerve appeared severely compromised. After neurolysis and removal of posteromedial osteophytes, the nerve was anteriorly transposed following excision of the medial intermuscular septum and wide release of the retinaculum and Osborne’s fascia between the two heads of the flexor carpi ulnaris (**c**). A double freestyle flap was required to provide full-length coverage of the nerve: the proximal portion was covered with a posteromedial freestyle flap supplied by branches of the recurrent ulnar artery, while the distal portion was reconstructed with an anteromedial freestyle flap based on collateral branches of the ulnar artery (**d**). Final intraoperative view showing definitive ulnar nerve coverage with the double flap (*) (**e**).

In patients with nerve lesions localized at the elbow, the skin incision was made along the previous surgical scar in 4 cases, while a posteromedial approach was used in 3 cases. Identification and exploration of the ulnar nerve were always the initial steps of the surgical procedure. The nerve was carefully freed from adhesions and scar tissue. In 4 cases, the nerve was transposed anteriorly; in these cases, the intermuscular septum was excised, the Osborne retinaculum was widely opened, and the fascia between the two heads of the flexor carpi ulnaris (FCU) was released. Once the nerve was fully decompressed and liberated from scar tissue, it was positioned in its definitive anatomical location. At this stage, the adipofascial flap was sculpted and customized according to the local tissue conditions (Figure 4). In 3 cases, the flap was based on perforator vessels arising from the posterior recurrent ulnar artery as described by Pagnotta in 2021 [18] (Figure 5), identified on the deep surface of the posteromedial subcutaneous tissue of the elbow. In the 4 cases where the nerve was transposed anteriorly, the flap was based on perforators arising from the proximal ulnar branch of the brachial artery. In one case, where the ulnar nerve was anteriorly transposed, a double freestyle flap was required to achieve full-length coverage; the proximal portion was covered with a posteromedial freestyle flap supplied by branches of the recurrent ulnar artery (Pagnotta flap), whereas the distal portion was reconstructed with an anteromedial freestyle flap based on collateral branches of the ulnar artery (Figure 6). In all cases, the nerve was then covered by the flap to improve the surrounding environment and to prevent the formation of perineural fibrosis. Particular attention was given to avoid kinking of the nerve and to ensure adequate protection of the nerve relative to the superficial cutaneous layer, thus preventing painful paresthesia triggered by Tinel’s sign.

**Figure 7:**
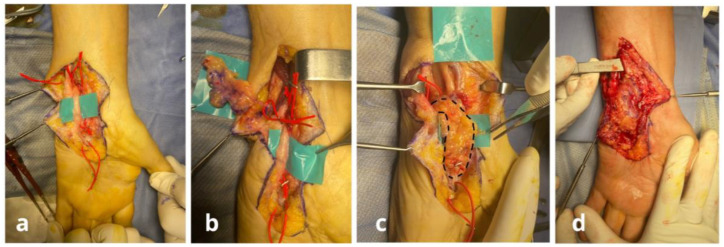
Case n°6. Intraoperative sequence of ulnar nerve reconstruction at the wrist. Exposure revealing a post-surgical ulnar nerve lesion (**a**). Elevation of a freestyle adipofascial flap based on collateral branches of the ulnar artery (**b**). Coverage of the ulnar nerve with the flap (**c**). Final aspect after tourniquet release, confirming adequate flap vascularization (**d**).

**Figure 8:**
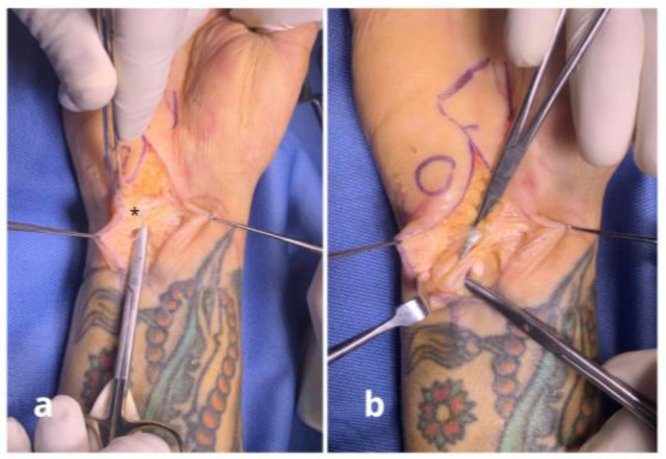
Case n°12 Right wrist with ulnar nerve neuropraxia following a displaced fracture, with volar dislocated ulnar fragments causing scar formation around the nerve (*) (**a**). After neurolysis, the nerve fascicles are visible, indicating severe nerve damage. The nerve was then covered with a local freestyle adipofascial flap (**b**).

In patients with nerve lesions localized at the wrist, a longitudinal skin incision was performed on the anterior ulnar aspect of the wrist and extended distally. Identification and careful dissection of both the ulnar artery and ulnar nerve were always the first steps of the procedure. The nerve was meticulously freed from adhesions and scar tissue. Guyon’s canal was opened in 6 cases. After decompression and release of the nerve from fibrotic and scarred tissue, it was repositioned in its anatomical bed. An adipofascial flap was then used to provide coverage. In 2 cases, the flap was based on perforators from the dorsal branch of the ulnar artery (modified Becker’s flap) (Figure 7). In 4 cases, a freestyle flap was based on random branches of the ulnar artery, as the dorsal branch had been injured during previous surgery or trauma. In all patients, the flap was laid over the nerve to optimize the perineural microenvironment and reduce the risk of fibrotic entrapment (Figure 8). Particular care was taken to preserve a tension-free course and to ensure adequate soft tissue coverage from the overlying skin, thereby minimizing the risk of painful paresthesia related to Tinel’s sign.

Both at the level of the elbow and wrist, the gliding, stability, and compression of the nerve were reassessed at the end of the procedure. The tourniquet was only released after confirming that the nerve was free from compression and properly covered and protected by the flap. Following tourniquet release, the vascular pedicle of the flap was carefully evaluated to ensure adequate flap perfusion before concluding the procedure. Finally, meticulous hemostasis was performed to minimize the risk of hematoma formation and perineural fluid collections, which could lead to complications such as infections or the development of perineural scar adhesions that may compromise surgical outcomes. Subcutaneous drains were placed in all patients.

## 3. Results

The mean follow-up period was 24.2 ± 12.1 (12–48) months. The mean age of patients was 49.3 ± 19 (18–88) years, with 7 males and 6 females. In 8 cases, the right upper limb (dominant side) was involved, while in 5 cases the left upper limb (non-dominant side) was affected. The mean preoperative Q-DASH score was 74.5 ± 7.75 (range: 90.9–59.1), while the mean postoperative Q-DASH score was 18.9 ± 11.46 (range: 52.3–9.1). The mean preoperative NRS score was 8.77 ± 0.83 (range: 10–7), whereas the mean postoperative NRS score at the last follow-up was 3.85 ± 1.46 (range: 2–6).

All patients demonstrated improvement in ulnar nerve–related symptoms, and the Tinel test along the ulnar nerve at the elbow and wrist was negative in all cases at the final follow-up.

The youngest patient (case no. 3), with post-traumatic elbow ankylosis and ulnar nerve pseudoparalysis, developed wound dehiscence at the posterior surgical site, necessitating a secondary operation with local skin flap reconstruction. Another patient (case no. 10), with severe elbow stiffness and a painful ulnar nerve neuroma, initially experienced a favorable postoperative course, with pain resolution and improved range of motion. However, three weeks after surgery, she developed a deep surgical site infection. The patient underwent a second surgical procedure involving extensive joint debridement to manage the infection. The previously transposed ulnar nerve was carefully evaluated during the procedure; the infection, which originated from the joint, did not appear to involve the nerve or the surrounding tissues. Therefore, the adipofascial flap previously performed was not revised. The patient completed a 6-week course of oral antibiotic therapy, resulting in full resolution of the infection. Nevertheless, a recurrence of elbow stiffness occurred—less severe than preoperatively. At the latest radiographic follow-up, clear signs of degenerative osteoarthritis of the elbow joint were observed. Despite this and despite a low Q-DASH score, the patient reported overall satisfaction with the outcome, particularly due to the significant improvement in ulnar nerve–related symptoms.

No additional complications were observed in our series. To date, no other patient has required further surgical intervention. All patients demonstrated clinical improvement in ulnar nerve–related symptoms, and Tinel’s sign along the course of the ulnar nerve at both the elbow and wrist was negative. Finally, all patients presenting with complex regional pain syndrome (CRPS) secondary to ulnar nerve injury achieved complete clinical resolution after neurolysis and vascularized adipofascial flap coverage of the nerve.

### Statistical Analysis

Statistical analysis was performed to compare pre- and postoperative NRS (pain) and Q-DASH (upper limb function) scores. Data distribution was assessed using the Shapiro–Wilk test: preoperative NRS (*p* = 0.012) and postoperative Q-DASH (*p* = 0.0016) scores were not normally distributed. Therefore, in addition to paired *t*-tests, the non-parametric Wilcoxon signed-rank test was applied. NRS scores decreased from a mean of 8.77 ± 0.83 preoperatively to 3.85 ± 1.46 postoperatively. This reduction was highly significant according to both the paired *t*-test (t = 10.43, *p* < 0.000001) and the Wilcoxon test (Z ≈ −3.06, *p* = 0.00024). Q-DASH scores improved from 74.5 ± 7.75 preoperatively to 18.9 ± 11.46 postoperatively. The difference was again highly significant (paired *t*-test: t = 14.01, *p* < 0.00000001; Wilcoxon: Z ≈ −3.06, *p* = 0.00024). These findings demonstrate a clinically and statistically significant improvement in both pain reduction and upper limb function following surgical treatment with the adipofascial flap.

## 4. Discussion

Traumatic injuries of the ulnar nerve are relatively frequent, although their reported incidence varies across studies [2,4,7,31,32,33,34,35]. A recent investigation identified the ulnar nerve as the most commonly affected peripheral nerve in upper limb trauma, with an estimated incidence of 3.86 cases per 100,000 persons per year [31]. Similarly, epidemiological data reported that peripheral nerve injuries occurred in approximately 1.3% of all trauma patients, with the ulnar nerve being the most frequently involved nerve in the upper extremity, particularly in lesions occurring between the elbow and the hand [32]. In a 2019 electrodiagnostic study, Raeissadat et al. found that 31.2% of neuropathies were trauma-related, with traumatic lesions more commonly affecting the forearm, while non-traumatic cases tended to involve the elbow [33]. In a retrospective study conducted by Mondelli and colleagues in 2004 on the incidence of ulnar nerve neuropathy in the province of Siena, 17.7% of cases were found to be post-traumatic (55 out of 311). In addition, 34 cases of ulnar neuropathy were attributed to recurrent elbow strain (10.9%) [36]. Peripheral ulnar neuropathies—whether resulting from trauma or surgery—remain a significant cause of upper limb dysfunction [4,7,9,37]. Mechanisms of injury include direct trauma such as laceration or traction, compression by hematomas or displaced bone fragments, and secondary complications like postoperative perineural scarring [1,2,3,4,5,6]. Although the association between ulnar nerve dysfunction and trauma to the elbow or wrist is well documented, many studies do not clearly differentiate between acute, iatrogenic, and delayed-onset neuropathies, limiting our ability to determine true prevalence rates [38].

In complex trauma scenarios—such as deep lacerations involving the ulnar side of the forearm, wrist, or hand—ulnar nerve involvement may occur in up to 90% of patients, as demonstrated in a recent 2023 retrospective analysis [3]. Additionally, ulnar nerve neuropathies may develop as iatrogenic complications of prior surgical procedures, particularly those involving the elbow, such as distal humerus fracture fixation, total elbow arthroplasty, or elbow arthroscopy [4,6,7,39,40,41,42,43]. In a prospective study of 1502 surgical patients, perioperative (surgery-related) ulnar neuropathy developed in 0.5% of cases, primarily in men aged 50–75 [7]. A 2019 systematic review and meta-analysis evaluating ulnar nerve complications following ulnar collateral ligament reconstruction (UCLR) in the elbow, encompassing 17 studies and 1518 patients, reported a high complication rate of approximately 12% [44]. In 2003, Little and colleagues published a review of all reported cases of total elbow arthroplasty available in the literature up to that time, which revealed an incidence of iatrogenic ulnar nerve injury of approximately 5% [45].

Severe ulnar nerve injuries often result in a combination of sensory and motor deficits. Sensory symptoms include numbness, paresthesia, or dysesthesia along the medial forearm, the fifth digit, and the ulnar half of the fourth digit. Patients may also experience burning pain, positive Tinel’s sign, and nocturnal discomfort that disrupts sleep. On the motor side, deficits typically include weakened grip and pinch strength, impaired fine motor coordination, and, in advanced cases, intrinsic muscle wasting leading to the classic “claw hand” deformity. These impairments significantly affect both function and quality of life [8,9,10]. When the nerve is severely damaged with complete loss of axonal continuity and disruption of its supporting structures (neurotmesis), external neurolysis alone is insufficient. In such cases, more advanced surgical strategies are necessary, such as direct neurorrhaphy, autologous nerve grafting, nerve transfers, neurotization procedures, or—when nerve function is irrecoverable—tendon transfers [46]. Conversely, in cases of neuropraxia or axonotmesis—where the structural integrity of the nerve is preserved but spontaneous recovery fails to occur—external neurolysis remains the most commonly performed and effective surgical approach [11,12,13,14]. The timing of surgical intervention in cases of nerve injury with preserved continuity or compressive neuropathy remains critical [5,47]. Some authors recommend an observation period of 2–3 months, during which clinical examination and electrodiagnostic studies are used to monitor for signs of spontaneous recovery [48]. If no improvement is noted, surgical exploration and neurolysis are typically indicated. However, careful consideration must be given to the potential complications of neurolysis, particularly the devascularization of the nerve and the formation of perineural adhesions, which can impede axonal regeneration and, in some cases, worsen neuropathic symptoms [16,20,25].

To address these issues, adipofascial flaps have been developed and described as a means of providing vascularized coverage to the nerve, protecting it during the healing phase and reducing the adhesion neurodesis effect. Aside from adipofascial flaps, several alternative strategies have been proposed to reduce postoperative Tinel’s sign and improve the regenerative milieu of peripheral nerves. Autologous vein wrapping and biologic or collagen-based nerve wraps act primarily as anti-adhesive barriers, and have shown symptomatic improvement in revision compression neuropathies, though they lack intrinsic vascularity. Umbilical cord and amniotic membranes provide similar protection with potential anti-inflammatory effects, but evidence remains limited. Conduits and processed nerve allografts are instead designed for gap reconstruction rather than coverage, with outcomes largely dependent on defect length. Compared with these options, the adipofascial flap provides a living, vascularized cushion that not only prevents recurrent scarring but also delivers trophic support, which may explain the consistent resolution of Tinel’s sign observed in our series [49,50,51,52,53].

Several authors have demonstrated the regenerative potential of adipose tissue when used as a flap to cover injured peripheral nerves [16,17,20,26,27,28]. The adipofascial flap, a relatively simple surgical procedure, creates an ideal microenvironment for nerve healing, particularly in posttraumatic cases complicated by soft tissue loss or scarring [16,20,21]. Although the literature on adipofascial flaps predominantly focuses on chronic compressive ulnar neuropathies or revision surgeries [54], their application in posttraumatic settings is equally relevant [25,29,30]. These flaps offer both mechanical protection and biological support, making them especially beneficial in high-risk cases where nerve regeneration may be compromised [17,18,20,22,23,24]. Hayashi et al. employed an ulnar recurrent adipofascial flap for one-stage reconstruction of massive defects around the elbow and forearm, achieving excellent surgical outcomes with no neurological complications observed [29]. Ramos and his group presented a series of twenty-two patients treated between 2012 and 2023for neurodesis and neuromas in continuity in the upper limb. They performed external neurolysis and adipofascial flap, achieving a statistically significant reduction in patients’ pain according to the VAS scale [30]. Between 2004 and 2010 Adani and his group treated 8 patients who had posttraumatic painful median nerve neuromas at the level of the wrist but with retained median nerve function with adipofascial flaps, achieving an average improvement in pain. Specifically, complete resolution of symptoms was obtained in 5 patients, a good improvement with mild residual pain in 2 patients, and no improvement in 1 patient, which was probably caused by excessive intraneural scarring [25].

Adipose tissue is not only a source of adipose-derived stem cells but also provides a highly vascularized environment that enhances nerve regeneration through paracrine signaling and the release of neurotrophic factors, including nerve growth factor (NGF), insulin-like growth factor-1 (IGF-1), neurotrophin-3 (NT-3), neurotrophin-4 (NT-4), and basic fibroblast growth factor (bFGF) [26,27,28]. Strickland et al. reported promising outcomes using hypothenar fat flaps in recurrent carpal tunnel syndrome [26]. Furthermore, adipose tissue has been shown to stimulate Schwann cells to produce endogenous neurotrophic factors such as brain-derived neurotrophic factor (BDNF) and glial cell line-derived neurotrophic factor (GDNF) [26,27,28]. Additional benefits of adipofascial flaps include the reduction in postoperative perineural fibrosis and protection of the nerve from minor external trauma [20]. Recent clinical case series further confirm these advantages [17,18,20,21].

Adipofascial flaps represent a valuable adjunct in the surgical management of ulnar nerve neuropathies, particularly in posttraumatic and postoperative cases involving the elbow and forearm. Their ability to promote nerve regeneration, reduce scarring, and protect neural structures highlights their role as an effective and reliable strategy in peripheral nerve reconstruction [16,20,21].

In our series, clinical outcomes were favorable across all treated cases. Even patients who experienced postoperative complications ultimately reported meaningful improvements, particularly in terms of neuropathic pain reduction—likely due to the protective and trophic effects of the adipofascial coverage on the ulnar nerve. One patient (n° 11), who developed a postoperative infection and did not achieve optimal functional recovery, nevertheless expressed satisfaction with the outcome, citing a substantial improvement in her preoperative neuropathic pain symptoms. This supports the hypothesis that extra care to the ulnar nerve through vascularized flap coverage may offer significant analgesic benefit in cases of chronic nerve suffering.

The statistical analysis of our series confirmed a significant improvement in both pain perception and upper limb function after surgical management with adipofascial flap coverage of the ulnar nerve. The reduction in NRS scores reflects a substantial alleviation of pain, while the marked decrease in Q-DASH scores demonstrates functional recovery and improved quality of life. Importantly, all patients showed resolution of preoperative Tinel’s sign, supporting the clinical relevance of the statistical findings.

To our knowledge, this is the first study to systematically evaluate the use of adipofascial flaps in the treatment of ulnar nerve pseudoparalysis and neuroma-in-continuity—not only at the elbow, where limited numbers of reports exist, but especially at the wrist level, where no previous case series have been published. This represents a major strength of our work and a novel contribution to the literature.

Our findings suggest that adipofascial flaps are a valuable adjunct in the surgical management of severe ulnar nerve injuries. The technique is relatively simple to perform, requiring only a few technical precautions—especially during flap dissection, where careful preservation of the vascular pedicle is essential. In our experience, the use of 2.5× magnification loupes greatly facilitates the identification and protection of the vascular structures, reducing the risk of iatrogenic damage.

However, this study has several limitations. The most significant is the small sample size, which limits the external validity of our results. Additionally, the heterogeneity of the clinical conditions treated could be considered a potential confounding factor. On the other hand, this variability may also be viewed as a strength: the consistent clinical improvement observed across a diverse range of indications highlights the technique’s adaptability and potential value in multiple surgical scenarios.

## 5. Conclusions

The surgical management of severe post-traumatic and post-surgical ulnar nerve neuropathies remains a challenging field. Our study suggests that personalized adipofascial flap coverage represents a safe, reproducible, and biologically favorable adjunct to standard external neurolysis procedures. Despite the small sample size and the heterogeneity of clinical presentations, the outcomes were encouraging, with improvement observed in functionality as well as significant pain reduction.

This technique, if properly executed, offers not only mechanical protection but also a regenerative microenvironment that may enhance axonal recovery and reduce the risk of perineural fibrosis. To our knowledge, this study represents the first attempt to clarify the role of adipofascial flaps in complex ulnar neuropathies involving both the elbow and wrist. Our findings support the utility of adipofascial flaps as a safe and biologically favorable adjunct in the surgical management of these challenging cases. Future prospective studies with larger series are mandatory to confirm these preliminary findings and to define optimal indications.

## Figures and Tables

**Table 1:** Clinical and demographic characteristics of patients included in the study.

Pt	Site	Diagnosis	Surgical Procedure	Previous Surgery	Pre-Op NRS	Pre-Op QDASH	Post-Op NRS	Post-Op QDASH	
1	Wrist	Postsurgical ulnar nerve neuroma	Nerve decompression + neurolysis + freestyle adipofascial flap	Wrist laceration with involvement of the ulnar nerve (open wound)	10	90.9	6	22.7	
2	Elbow	Post-surgical ulnar nerve pseudo-palsy following total elbow arthroplasty	Nerve decompression + neurolysis + Pagnotta’s adipofascial flap	Oncology elbow resection and total elbow arthroplasty (TEA)	8	72.7	5	25.0	
3	Elbow	Post-surgical elbow stiffness and ulnar nerve pseudo-palsy	Hardware removal + arthrolysis + ulnar nerve anterior transposition + freestyle anterior adipofascial flap	Distal humerus open reduction and internal fixation (ORIF)	9	68.2	3	11.4	
4	Wrist	Post-surgical ulnar nerve pseudo-palsy and complex regional pain syndrome (CRPS)	Nerve decompression + neurolysis + freestyle adipofascial flap	Neurorrhaphy and FCU repair (open wound)	9	70.5	2	13.6	
5	Wrist	Post-surgical ulnar nerve neuroma and complex regional pain syndrome following distal radius fracture treated with osteosynthesis and wrist CRPS	Nerve decompression + neurolysis + adipofascial flap based on dorsal ulnar artery (DUA)	Distal radius open reduction and internal fixation (ORIF)	9	81.8	2	13.6	
6	Wrist	Post-surgical ulnar nerve palsy and wrist CRPS	Nerve decompression + neurolysis + adipofascial flap based on dorsal ulnar artery (DUA)	Neurorrhaphy and FCU repair (open wound)	10	70.5	3	22.7	
7	Elbow	Ulnar nerve pseudo-palsy in multiple hereditary exostoses,	Ulnar nerve decompression + anterior transposition + double adipofascial flap	None	8	72.7	4	15.9	
8	Elbow	Post-surgical ulnar nerve neuropathy in post-traumatic osteoarthritis	Ulnar nerve decompression + anterior transposition + free style anterior adipofascial flap + (TEA)	Open debridement and in situ ulnar nerve neurolysis	9	59.1	5	22.7	
9	Elbow	Post-traumatic ulnar nerve neuropathy and elbow stiffness following elbow dislocation	Nerve decompression + neurolysis + Pagnotta’s adipofascial flap	None	9	75.0	5	15.9	
10	Elbow	Post-surgical ulnar nerve neuropathy	Hardware removal + arthrolysis + ulnar nerve anterior transposition + freestyle anterior adipofascial flap	Distal humerus ORIF	9	75.0	6	52.3	
11	Elbow	Post-traumatic ulnar nerve neuropathy and elbow stiffness following radial head fracture dislocation	Arthroscopic arthrolysis + nerve decompression + Pagnotta’s adipofascial flap	None	7	77.3	4	9.1	
12	Wrist	Post-traumatic ulnar nerve neuropathy following distal radius fracture malunion with fragment impinging on the ulnar nerve	Ulnar bony fragment removal + distal radius osteotomy + ulnar nerve neurolysis + freestyle adipofascial flap	None	8	81.8	2	11.4	15
13	Wrist	Post-surgical ulnar nerve neuropathy following K-Wires penetration	Distal radius osteotomy + ulnar nerve neurolysis + freestyle adipofascial flap	Distal radius osteosynthesis with plate and K-wires	9	79.5	3	9.1	24

(CRPS) complex regional pain syndrome; (ORIF) open reduction and internal fixation; (DUA) dorsal ulnar artery; (FCU) flexor carpi ulnaris; (TEA) total elbow arthroplasty.

## Data Availability

The data presented in this study are available on reasonable request from the corresponding author due to patient privacy.
